# The Interactome in the Evolution From Frailty to Sarcopenic Dependence

**DOI:** 10.3389/fcell.2021.792825

**Published:** 2021-12-02

**Authors:** Ana Coto-Montes, Laura González-Blanco, Eduardo Antuña, Iván Menéndez-Valle, Juan Carlos Bermejo-Millo, Beatriz Caballero, Ignacio Vega-Naredo, Yaiza Potes

**Affiliations:** ^1^ Department of Cell Biology and Morphology, Faculty of Medicine, University of Oviedo, Oviedo, Spain; ^2^ Instituto de Investigación Sanitaria del Principado de Asturias (ISPA), Av. del Hospital Universitario, Oviedo, Spain; ^3^ Instituto de Neurociencias del Principado de Asturias (INEUROPA), University of Oviedo, Oviedo, Spain; ^4^ Área de Sistemas de Producción Animal, Servicio Regional de Investigación y Desarrollo Agroalimentario (SERIDA), Villaviciosa, Spain

**Keywords:** frailty, dependence, sarcopenia, skeletal muscle, interactome, biomarkers, autophagy

## Abstract

Biomarkers are essential tools for accurate diagnosis and effective prevention, but their validation is a pending challenge that limits their usefulness, even more so with constructs as complex as frailty. Sarcopenia shares multiple mechanisms with frailty which makes it a strong candidate to provide robust frailty biomarkers. Based on this premise, we studied the temporal evolution of cellular interactome in frailty, from independent patients to dependent ones. Overweight is a recognized cause of frailty in aging, so we studied the altered mechanisms in overweight independent elderly and evaluated their aggravation in dependent elderly. This evidence of the evolution of previously altered mechanisms would significantly support their role as real biomarkers of frailty. The results showed a preponderant role of autophagy in interactome control at both different functional points, modulating other essential mechanisms in the cell, such as mitochondrial capacity or oxidative stress. Thus, the overweight provoked in the muscle of the elderly an overload of autophagy that kept cell survival in apparently healthy individuals. This excessive and permanent autophagic effort did not seem to be able to be maintained over time. Indeed, in dependent elderly, the muscle showed a total autophagic inactivity, with devastating effects on the survival of the cell, which showed clear signs of apoptosis, and reduced functional capacity. The frail elderly are in a situation of weakness that is a precursor of dependence that can still be prevented if detection is early. Hence biomarkers are essential in this context.

## 1 Introduction

Although aging is usually associated with dependence and disease, approximately 50% of the elderly report in different health surveys that they are in good or very good health (SHARE, 2021), which is usually associated with the absence of important symptoms of possible existing chronic diseases and the absence of disabling diseases (Mearin et al., 2009). However, with the increase in life expectancy, this percentage of elderly people free of dependency is rapidly decreasing, with serious health, social, and economic consequences. It is therefore necessary to develop preventive policies and treatments to delay the onset of such dependence and multimorbidity (Coto-Montes et al., 2016). Clinically, frailty is defined as a multifactorial state, dependent on aging, which increases the individual’s vulnerability to disease, dependence, and death (Fried et al., 2001). The detection of this stage prior to dependence would allow us to act on it preventively, delaying the onset of dependence (de Gonzalo-Calvo et al., 2012). There are many factors that collaborate in the development of frailty and, interacting with each other, end up causing this situation of vulnerability. Telomeres have been widely described to shorten with age, so the telomere length is considered as a hallmark of the aging process and frailty development affecting cell signaling pathways (Lorenzi et al., 2018). Moreover, oxidative stress, inflammation, hypertension, hyperlipidemia… are factors widely recognized as being involved in the triggering of frailty. Together with them, sarcopenia, the loss of muscle capacity and quality associated with aging, is one of the processes that shares the most characteristics with frailty, so that its study and the knowledge of the factors involved in its development could provide, in parallel, information on the progression from frailty to dependence in the elderly. But sarcopenia, like frailty, is a multifactorial process in which multiple alterations, most of them at the muscle fiber level, progressively induce cell failure and reduce the muscle strength and power that characterizes sarcopenia. The fact that these alterations occur simultaneously implies an interrelation that must also be taken into account in the assessment of the situation, which forces us to study the cellular interactome in these circumstances.

The cellular interactome is a term that refers specifically to the interactions established between the various organelles of the cell, through vesicular and molecular relationships. This process, widely accepted at the physiological level, is rarely studied at the pathological level when, nevertheless, the concatenation of damage is inevitable in such a controlled and closely controlled environment as the cellular one. A recent review summarizes a possible correlation of telomere length at the systemic level with frailty and sarcopenia. However, it is questioned whether it could be considered an age estimation of skeletal muscle which is a postmitotic tissue (Lorenzi et al., 2018). Therefore and given the great importance of generating personalized therapies and treatments that involves specifical biomarkers, the study of the cellular interactome is decisive. The study of the interactome, which requires the study of the capacity of action of the main cellular organelles, as well as the response mechanisms that the cell uses to try to solve the functional alterations and thus avoid cell death, favors the identification of biomolecules that stand out for their importance in the affected processes and that could have an important role as biomarkers.

Although the concept of biomarker as such has existed since the very beginnings of medical practice, the use of laboratory-developed biomarkers for clinical research is more novel and presents important gaps in its definition in terms of the functions that such biomarkers can perform (Strimbu and Tavel, 2010). Thus, although in 2001, the National Institutes of Health Biomarkers Definitions Working Group defined biomarker as an indicator of physiological processes, pathological processes or response to pharmacological agents (Strimbu and Tavel, 2010), in the same year the World Health Organization (WHO) in coordination with the United Nations and the International Labor Organization, proposed another definition with a substantial change with respect to the previous one. Thus, WHO defines biomarker as any measurable substance, process or structure from the body or any product there of that influences or predicts the incidence of disease (WHO International Programme on Chemical Safety, 2001). The inclusion of the ability to predict outcomes in the definition of biomarker opened up infinite possibilities while at the same time greatly complicated the concept. A descriptive biomarker immediately shows its ability to confirm the repeated process. A predictive biomarker, however, is a molecule of the present that can only confirm its effectiveness in the future, which makes its discovery and confirmation of its ability difficult. This future confirmation of its success as a biomarker is rarely shown in the article, so only reviews that include information from several articles are able to confirm or discard the predictive capacity of the biomarker.

The lack of adequate information to detect, at currently, frail people, leads to the fact that the study of this situation of vulnerability must be developed in models, whether animal or human, from whose study we can learn the basis of this possible lack of protection and detect its markers (Rutenberg et al., 2018). Thus, for instance, the well-known deleterious effects that obesity causes at the organic level, the drastic decrease in the life expectancy of obese people and their low representativeness in aging, allow us to assume that overweight, but not obesity because it is directly disabling, could be a good model to induce frailty in the elderly.

For all these reasons, the detection of frailty in the elderly that, in a predictive manner, induces the delay or disappearance of dependence in aging is one of the main objectives of geriatrics today. To achieve this, it is necessary to have predictive biomarkers obtained from a frailty model. If we take into account that sarcopenia shares with frailty a large number of common processes, if we could study the evolution of sarcopenia from an incipient to a deleterious phase, we could also know the evolution of these selected biomarkers and finally confirm their predictive capacity. The problem arises in the detection of this incipient and therefore not yet incapacitating phase of sarcopenia. For this purpose, the introduction of the variable “overweight” without performing any physical activity since it improves muscle capacity (Giovannini et al., 2010), is presented as the ideal condition due to its potential to induce affectation (Benton et al., 2011; Atkins et al., 2014). Thus, the present review is focused in the study of the data published so far showing sarcopenic evolution from independent to highly dependent elderly people, providing with powerful contrasted biomarker predictors.

## 2 Predictive Biomarkers of Sarcopenic Dependence

Although all organelles are essential for proper cellular functioning whatever their function, it is evident that, in the case of muscle fibers, organelles such as the rough endoplasmic reticulum (ER), responsible for protein synthesis (Deldicque, 2013) and mitochondria for their role in energy production (Chanseaume et al., 2010) stand out for their importance for muscle activity. In the same way, energy metabolism collaborates significantly in the maintenance of protein balance and skeletal muscle mass (Pugh et al., 2013) and autophagy is foreseen as the essential mechanism for the maintenance of adequate survival conditions (Rashid et al., 2015; García-Prat et al., 2016a; García-Prat et al., 2016b), preventing the triggering of apoptosis, even at the cost of reducing muscle capacity (Potes et al., 2017). Therefore, these organelles and mechanisms should be the primary targets of our study of the interactome. On the other hand, the assessment of the possible limitations that condition aging makes it advisable to include myogenesis (Morgan and Partridge, 2003; Cerletti et al., 2012) and myogenic regulatory factors (MRFs) (Sousa-Victor et al., 2014; Baraibar et al., 2016) as secondary targets.

The studies developed on the basis of the indicated premises showed that the selected model of overweight proved to be an imperceptible, but potent inducer of sarcopenia in independent elderly belonging to the HIPA cohort (Charlson et al., 1987; Caballero et al., 2014; Bermúdez et al., 2018), involving all the targets selected in the study that suggested a significant alteration of the interactome (Potes et al., 2017; Potes et al., 2019).

ER-stress becomes evident when misfolded and unfolded proteins are significantly increased and promotes the unfolded protein response (UPR) which manifests in three independent response cascades named after the initiating ER transmembrane protein in each case: inositol-requiring protein 1 (IRE-1), activating transcription factor 6 (ATF-6) and RNA-dependent protein kinase like ER eukaryotic translation initiation factor 2 alpha kinase (PERK) (Schröder and Kaufman, 2005). Each of them induces a cascade of responses directly related to the severity of involvement. In the case of the effect of overweight, a recent review demonstrated that a high-fat diet leads to a disruption of calcium homeostasis with both effects being clearly correlated with increased ER stress (Deldicque, 2013), so that the observed IRE-1 and ATF-6 cascade firing in independent overweight elderly was amply justified. Triggering of IRE-1 was associated with an inhibition of insulin receptor signaling by the serine phosphorilation of insulin receptor substrate-1 (IRS-1) that usually leads to insulin resistance, which is undoubtedly a marker of adverse prognosis because of its association with multiple comorbidities in aging (Hirosumi et al., 2002). Stimulation of this pathway has also been related to increased protein degradation (Mei et al., 2013), of special interest in the maintenance of muscle fibers.

Skeletal muscle has a high energetic demand, necessary for the conservation of its mass and protein balance (Koopman et al., 2014; Potes et al., 2019), therefore, it has three major energy metabolic systems to produce energy, in addition to mitochondria: phosphagen system, glycogenolysis and glycolysis pathways. All three showed a significant reduction of their activity in overweight independent individuals (Potes et al., 2019), even though there is a large literature showing the significant deleterious effects on muscle of this reduction (van Deursen et al., 1994; Gelfi et al., 2006; O’Connell et al., 2007; O’Connell et al., 2008) leading to rapid muscle senescence. Overweight also induced important alterations in the mitochondrial machinery with abrupt reductions in the expression of the main components of the electron transport chain and reduced fusion and fission capacities, indicating an imbalance in mitochondrial dynamics and mitochondrial dysfunction (Potes et al., 2019), which made it a candidate for mitophagy (Palikaras et al., 2015a; Palikaras et al., 2015b).

Autophagy due to its ability to recycle misfolded proteins is an essential mechanism in the fight against ER stress and some UPR pathways trigger it (Rashid et al., 2015). Autophagy is also an important regulator of insulin homeostasis, relying on its ability to carry out catabolic processes that regulate insulin production (Riahi et al., 2016). The appropriate induction of autophagy through the regulation of AMP-activated protein kinase (AMPK), the phosphatidylinositol-3-kinase (PI3K)/Akt and the mammalian target of rapamycin (mTOR) signaling pathways seems to suppress skeletal muscle loss (Bonaldo and Sandri, 2013; Fan et al., 2016). AMPK activates the autophagy-initiating serine/threonine protein kinase ULK1 (ULK1), whereas the PI3K/Akt/mTOR axis acts as a negative regulator of autophagy by blocking the activation of ULK1. Several studies have shown a clear effect of defective autophagy on muscle under conditions of obesity, where reduced activation of AMPK and increased induction of AKT and mTOR were found (Turpin et al., 2009; Kirk-Ballard et al., 2014; Barlow and Thomas, 2015; Ryu et al., 2020). However, the interplay between overweight and autophagy and its implication on muscle wasting are far from fully understood. The addition of the aging variable to this difficult relationship, in which an evident ER stress has already been shown, could favor the progression of age-related atrophy or development of overweight-associated diseases in the aged population. The results obtained in muscle biopsies of overweight elder people showed increased levels of the phosphatidylethanolamine-conjugated form of LC3 (LC3-II),which is essential for autophasome formation, together with high expression levels of sequestosome 1 or p62 and neighbor of BRCA1 gene 1(NBR1), autophagic receptors for the degradation of ubiquitinated protein aggregates (Kirkin et al., 2009; Katsuragi et al., 2015). These data suggest the accumulation of autophagosomes and the possible alteration in the last phases of autophagy (Potes et al., 2017). Autophagy is usually defective and slow in aging, characterized by decreased levels of AMPK, increased activation of mTOR and lower levels of LC3-II (Sandri et al., 2013; Bujak et al., 2015; García-Prat et al., 2016b). This fact suggests that autophagy is overwhelmed in the elderly and overweight requires a degree of autophagy that aging does not allow. Previous studies carried out under conditions of high autophagy demand showed that the maintenance of these extreme conditions resulted in significant cellular damage (García-Macia et al., 2014). All this suggests that the excessive demand placed on independent elderly people who are overweight cannot be maintained for a long time, increasing its alteration proportionally to the demand, which in a short period of time will cause irreversible damage to autophagy and, with it, a significant increase in cellular damage. If so, and if the role of overweight as an inducer of fragility is confirmed, autophagy would be revealed as an essential biomarker predicting dependence.

In view of the previously showed results, the reduction in the activity of MRFs observed in overweight seniors was clearly to be expected, as autophagy plays an essential role in the maintenance of stemness and the prevention of senescence (García-Prat et al., 2016a). This significant reduction in Myf5 and Myf6 together with the observed stability in myostatin, a negative regulator of skeletal muscle growth (Yablonka-Reuveni, 2007) could contribute significantly to muscle atrophy and degeneration.

The cellular alterations described in overweight but highly independent elderly people would indicate a high predisposition, in the near future, to the development of an exacerbated sarcopenia that would provoke the dependence we wish to avoid. That is why the molecules detected, and fundamentally those indicative of autophagy, due to their catalytic role in the rest of the impariments, would be powerful biomarkers predictive of dependence and indicators of frailty. But this could only be confirmed by studying the evolution of these subjects until they reach dependence, which means that articles showing the muscular interactome of dependent elderly subjects displaying exacerbated sarcopenia acquire essential importance in the reaffirmation of the previous diagnosis of these markers.

Dependent individuals over 70 years of age and from the same HIPA cohort (Bermúdez et al., 2018) from which the independent control and overweight individuals were previously extracted for the study of their interactome (Potes et al., 2017; Potes et al., 2019) were studied in order to confirm the hypotheses raised on the evolution of the observed alterations. Results from dependent elderly showed serious alterations in their cellular interactome at the skeletal muscle level, confirming the development of an exacerbated sarcopenia. Protein synthesis was already inefficient, ER activity was also severely compromised and the UPR pathway remained activated. The abrupt reduction in overall energy production in the cell, together with the evident mitochondrial impairment revealed series of compromises in all energy-dependent activities in the cell. Interestingly, skeletal muscle biopsies from dependent elderly also exhibited altered autophagic response characterized by reduced LC3-II and p62 levels. These data together with those previously obtained in overweight elder people suggest that there is not a decrease in autophagic activity during sarcopenic evolution from independent to highly dependent elderly people, but a blockage of autophagic response. The death of highly damaged cells should not necessarily cause damage to the muscle as long as the regenerative capacity remains unaltered. But, as expected from the scenario shown, the activity of MRFs was drastically reduced, while a significant increase in myostatin synthesis was observed. Based on the data presented, muscle degeneration and atrophy accompany the irreversible loss of muscle mass which could be, by itself, the cause of dependence in the individuals studied due to the development of exacerbated sarcopenia (González-Blanco et al. under peer review).

## 3 Conclusion and Perspectives

**FIGURE 1:**
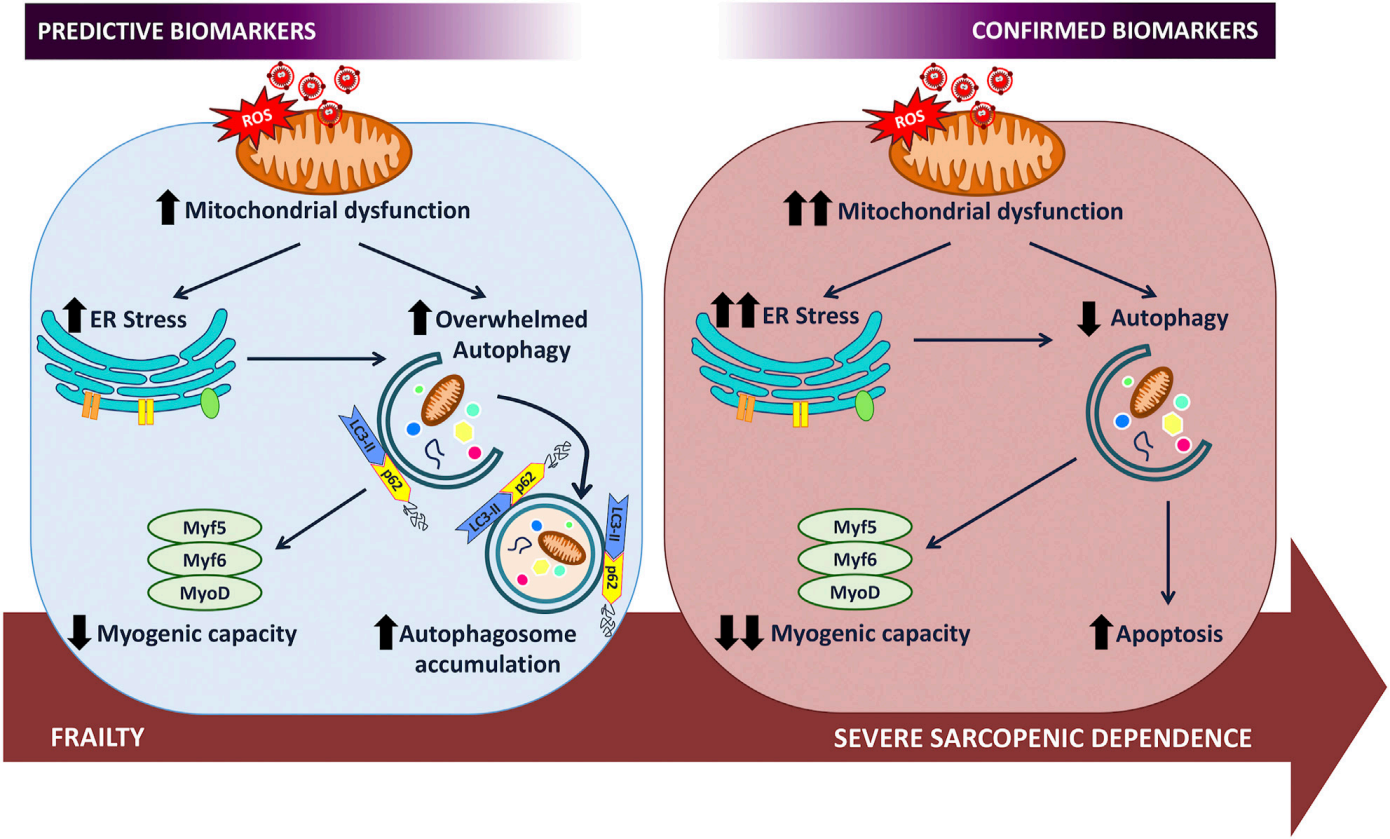
The study of the evolution of the frailty interactome that determines predictive biomarkers of sarcopenic dependence.

Only temporal studies will show us the evolution of the mechanisms selected within the cellular interactome and will be able to provide sufficient information to be able to choose among all the variables studied those that have a clear relevance in the deterioration of the organ to be studied and that can, therefore, reach the category of predictive biomarkers. In the case of the muscle of the elderly, the data presented show that autophagy plays an essential role in the evolution from fragility, modeled by overweight, to dependence, as a mechanism that exacerbates its activity in fragility to maintain the homeostatic balance of the cell. Its failure due to exhaustion leads the cell to a concatenation of energetic and functional alterations such that it ends up provoking apoptosis in conditions of loss of regenerative capacity (Figure 1). Consequently, autophagic markers and MRFs are important biomarkers of frailty as well as predictors of dependence and, therefore, therapeutic targets on which to direct preventive treatments that protect them and thus favor the delay of dependence.
